# Antibiotic resistance is linked to carriage of *papC* and *iutA* virulence genes and phylogenetic group D background in commensal and uropathogenic *Escherichia coli* from infants and young children

**DOI:** 10.1007/s10096-016-2854-y

**Published:** 2016-12-06

**Authors:** N. Karami, A. E. Wold, I. Adlerberth

**Affiliations:** 1 Department of Infectious Diseases, Institute of Biomedicine, The Sahlgrenska Academy, University of Gothenburg, Gothenburg, Sweden; 2 Department of Clinical Microbiology, University of Gothenburg, Guldhedsgatan 10A, 413 46 Göteborg, Sweden

## Abstract

P fimbriae, enabling adherence to colonic and urinary epithelium, and aerobactin, an iron sequestering system, are both colonization factors in the human colon and virulence factors for urinary tract infection. The colonic microbiota is suggested to be a site suitable for the transfer of antibiotic resistance genes. We investigated whether phenotypic resistance to antibiotics in commensal and uropathogenic *Escherichia coli* from infants and young children is associated with carriage of virulence genes and to phylogenetic group origin and, in the case of fecal strains, to persistence in the gut and fecal population levels. The commensal strains (*n* = 272) were derived from a birth cohort study, while the urinary isolates (*n* = 205) were derived from outpatient clinics. Each strain was assessed for phenotypic antibiotic resistance and for carriage of virulence genes (*fimA*, *papC*, *sfaD*/*E*, *hlyA*, *iutA*, *kfiC*, and *neuB*), phylogenetic group (A, B1, B2, or D), and markers of particular virulent clones (CGA-D-ST69, O15:H1-D-ST393, and O25b:H4-B2-ST131). Resistance to ampicillin, tetracycline, and trimethoprim was most prevalent. Multivariate analysis showed that resistance to any antibiotic was significantly associated with carriage of genes encoding P fimbriae (*papC*) and aerobactin (*iutA*), and a phylogenetic group D origin. Neither fecal population numbers nor the capacity for long-term persistence in the gut were related to antibiotic resistance among fecal strains. Our study confirms the importance of phylogenetic group D origin for antibiotic resistance in *E. coli* and identifies the virulence genes *papC* and *iutA* as determinants of antibiotic resistance. The reason for the latter association is currently unclear.

## Introduction

*Escherichia coli* is the leading cause of urinary tract infection (UTI), which frequently occurs in infants and young children [1]. Strains causing UTI usually originate in the patient’s own colonic microbiota [2]. *Escherichia coli* strains segregate into four major phylogenetic groups, termed A, B1, B2, and D. Most strains causing UTI are found within group B2, and to a lesser extent group D, while most strains belonging to the A and B1 phylogenetic groups have low pathogenic potential [3]. Further, a majority of uropathogenic *E. coli* strains express P fimbriae, which mediates adherence to colonic and urinary tract epithelium, the iron sequestering aerobactin system, and certain O and K antigens. However, these traits are also associated with superior capacity to colonize the human colon. Thus, *E. coli* strains that persist for long periods in the commensal microbiota more often than transient strains belong to group B2 [4], have O and K antigens characteristic of uropathogenic strains [5], and carry the genes for P fimbriae and aerobactin [6, 7]. In a rodent model, P fimbriae and capsule K5 confer the capacity to colonize the intestinal tract, suggesting that known UTI virulence traits in *E. coli* might primarily serve to increase fitness in the natural niche, the colon [8, 9]. The colonic microbiota has been suggested to be a milieu that favors transfer of antibiotic resistance genes from resistant to initially susceptible strains, and fitness in the human gut commensal microbiota might enhance the probability of acquisition of resistance genes [10]. Furthermore, colonization of the human gut would increase the likelihood of the strains being exposed to antibiotics used in human clinical practice, which would tend to positively select for antibiotic-resistant strains in the microbiota.

Certain uropathogenic *E. coli* clones are known to have accumulated antibiotic resistance genes, for example, clone O25b:H4-B2-ST131 [11] that frequently carries genes encoding extended-spectrum beta-lactamase (ESBL) of the CTX-M-15 type [12], clone CGA-D-ST69 that is globally spread and often carries genes conferring resistance to ampicillin, chloramphenicol, streptomycin, sulfonamides, tetracycline, and trimethoprim [13, 14], and clone O15:H1-D-ST393 that has acquired various antibiotic resistance genes over the last several decades [15]. The prevalence of these clones among commensal *E. coli* is unknown.

The aim of the present study was to investigate whether resistance to commonly used antibiotics is related to phylogenetic group, markers of virulence, and fitness in the commensal gut microbiota. Fecal and urinary *E. coli* isolates from Swedish infants were screened for antibiotic resistance and their virulence gene carriage, clonal origin, and phylogenetic group allocation were analyzed. Uropathogenic clones with known accumulation of antibiotic resistance genes were identified. A part of the fecal strains had previously been characterized regarding fecal population counts and capacity for long-term persistence in the microbiota [6]. Our results indicate that certain virulence traits are associated with an antibiotic-resistant phenotype.

## Materials and methods

### Commensal fecal *E. coli* strains from Swedish infants

In total, 272 commensal fecal *E. coli* strains isolated during 1998–2002 from 128 Swedish 0–1-year-old infants included in the birth cohort ALLERGYFLORA were studied. ALLERGYFLORA was designed to investigate the impact of gut microbiota on allergy development and the methodology has been described previously [16]. In brief, rectal swabs obtained on day 3 were cultured semi-quantitatively and fecal samples obtained at 1, 2, 4, and 8 weeks and at 6 and 12 months of age were cultured quantitatively on Drigalski agar [16]. Isolates were speciated using the API 20E biotyping system (bioMérieux, Marcy-l’Etoile, France) and *E. coli* were strain-typed using random amplified polymorphic DNA and analyzed regarding virulence gene carriage and phylogenetic group allocation [4, 6]. In each fecal sample, the counts of each strain [colony-forming units (CFU)/g feces] was determined [16]. In addition, for 70 of the 128 infants, each strain has previously been characterized as resident or transient in the microbiota of the infant, the former defined as colonizing for at least 3 weeks, the latter for a shorter period [17], and the *E. coli* colonization pattern of these 70 infants has been reported [6].

Each of the 272 strains identified were included only once in each analysis, even if it was isolated from an infant on several sampling occasions.

### Urinary *E. coli* isolates from Swedish infants

A total of 205 community-acquired urinary *E. coli* isolates from 0–2-year-old children sampled in November 2002–February 2003 or March 2004–August 2005 were included. Consecutive urinary samples cultured at the Clinical Microbiology Laboratory, Sahlgrenska University Hospital and yielding *E. coli* as the primary pathogen were included providing that: (1) they were derived from pediatric outpatients’ clinics or the emergency ward at the Queen Silvia Children’s Hospital in Gothenburg or (2) were from children aged 0–2 years old who had not yielded a previous positive urinary culture or been hospitalized prior to the positive sample. Isolation and speciation of *E. coli* were done using routine methods. For this study, each speciation was confirmed by using API 20E biotyping (bioMérieux).

### Virulence genotyping, phylogenetic group typing, and clonal identification

All urinary isolates and 129 fecal *E. coli* strains that had previously not been analyzed were screened for carriage of *fimA*, *papC*, *sfaD*/*E*, *neuB*, *kfiC*, *iutA*, and *hlyA*, as previously described [6].

Assignment to one of the phylogenetic groups A, B1, B2, or D was done using a previously described triplex polymerase chain reaction (PCR) method [18].

All phylogenetic group D strains were screened for the clones CGA-D-ST69 and O15:H1-D-ST393 [19–21]. Phylogenetic group B2 strains were screened to identify the O25b:H4-B2-ST131 clone [22]. The *E. coli* strains CCUG 55212, CCUG 41424, and CCUG 61908 were used as reference strains.

### Antibiotic susceptibility

Phenotypic resistance to antibiotics was assessed using the agar disk diffusion method as described by the Nordic Committee on Antimicrobial Susceptibility Testing (NordicAST; http://www.nordicast.org). The following antibiotic disks were used: ampicillin, cefuroxime, cefoxitin, mecillinam, cefadroxil, ceftazidime, chloramphenicol, gentamicin, tobramycin, nitrofurantoin, nalidixic acid, tetracycline, and trimethoprim (Oxoid Ltd., Hants, UK). Strains belonging to the CGA-D-ST69 clone were also tested using disks containing streptomycin and sulfonamide. The antibiotic disks were applied on agar plates seeded with bacteria and the plates were incubated aerobically at 37 °C for 16–24 h. Thereafter, the zone diameters were measured and the strains were defined as sensitive or resistant according to the breakpoints provided by NordicAST.

At least the first isolate of each strain (*n* = 272) was tested for phenotypic resistance to antibiotics, and the resistance pattern of the first isolate of each strain was used in the analyses.

### Statistical methods

Proportions were compared using Fisher’s exact test (GraphPad Prism; GraphPad Software, La Jolla, CA, USA). The data were analyzed by multiple linear regression (SPSS version 21 for Windows; SPSS Inc., Chicago, IL, USA) to assess the independent association of different factors with antibiotic resistance in *E. coli*.

## Results

### Antibiotic resistance in fecal and urinary *E. coli* strains

Twenty-one percent of the commensal fecal strains and 40% of the urinary strains were resistant to at least one antibiotic (*p* < 0.0001). Resistance against ampicillin, tetracycline, and trimethoprim were most prevalent and more common among UTI than fecal strains (*p* ≤ 0.002, Table 1).

**Table 1:** Prevalence of resistance to antibiotics among fecal and urinary *Escherichia coli* strains deriving from Swedish infants

	Resistance prevalence				
	Fecal strains (*n* = 272)		Urinary isolates (*n* = 205)		*p*-Value
	*n*	%	*n*	%	
Ampicillin	32	12	60	29	<0.0001
Tetracycline	28	10	42	20	0.002
Trimethoprim	21	8	38	19	0.0006
Chloramphenicol	3	1	17	8	0.0001
Nalidixic acid	2	1	6	3	0.08
Nitrofurantoin	1	0.5	0	0	
Gentamicin	0	0	2	1	
Mecillinam	0	0	1	0.5	
Any of the above antibiotics	57	21	82	40	<0.0001

Fecal *E. coli* strains derived from 128 infants followed during the first year of life with regular fecal sampling. Individual strains were identified by random amplified polymorphic DNA [6]. UTI *E. coli* isolates were consecutive isolates obtained from positive urinary samples of children below 2 years of age presenting with UTI at pediatric outpatient clinics or the emergency ward at the regional children’s hospital. All isolates were tested by disk diffusion for resistance to the following antibiotics: ampicillin, cefuroxime, cefoxitin, mecillinam, cefadroxil, ceftazidime, chloramphenicol, gentamicin, tobramycin, nitrofurantoin, nalidixic acid, tetracycline, and trimethoprim. Only *p*-values below 0.10 are presented (Fisher’s exact test)

Urinary strains were isolated somewhat later (2002–2005) than the fecal strains (1998–2002). However, resistance rates in neither of these strain collections increased over the sampling period (data not shown). Furthermore, urinary strains isolated in 2002 (*n* = 63) were significantly more resistant than fecal strains collected during this year (*n* = 20) (*p* = 0.007). Another potential source of confounding was that commensal strains were isolated from 0–1-year-old infants, while urinary strains derived from 0–2-year-old infants. However, when comparing commensal strains isolated from 0–1-year-old infants with UTI isolates from infants of the same age, resistance was significantly higher in UTI isolates than commensal strains, 21 vs. 42%, *p* < 0.0001. Further, resistance among UTI isolates was not significantly different between isolates from 0–1-year-old infants compared with those from 1–2-year-old infants, 42 vs. 31%, *p* = 0.26.

### Antibiotic resistance and phylogenetic lineages

Uropathogenic *E. coli* strains mostly belong to phylogenetic groups B2 or D. Accordingly, a larger fraction of urinary compared to fecal strains belonged to phylogenetic groups B2 (72 vs. 46%, *p* < 0.0001) and D (20 vs. 14%, *p* = 0.11). Instead, more fecal than urinary strains belonged to phylogenetic groups A (28 vs. 4%, *p* < 0.0001) and B1 (12 vs. 4%, *p* = 0.002).

**Fig. 1:**
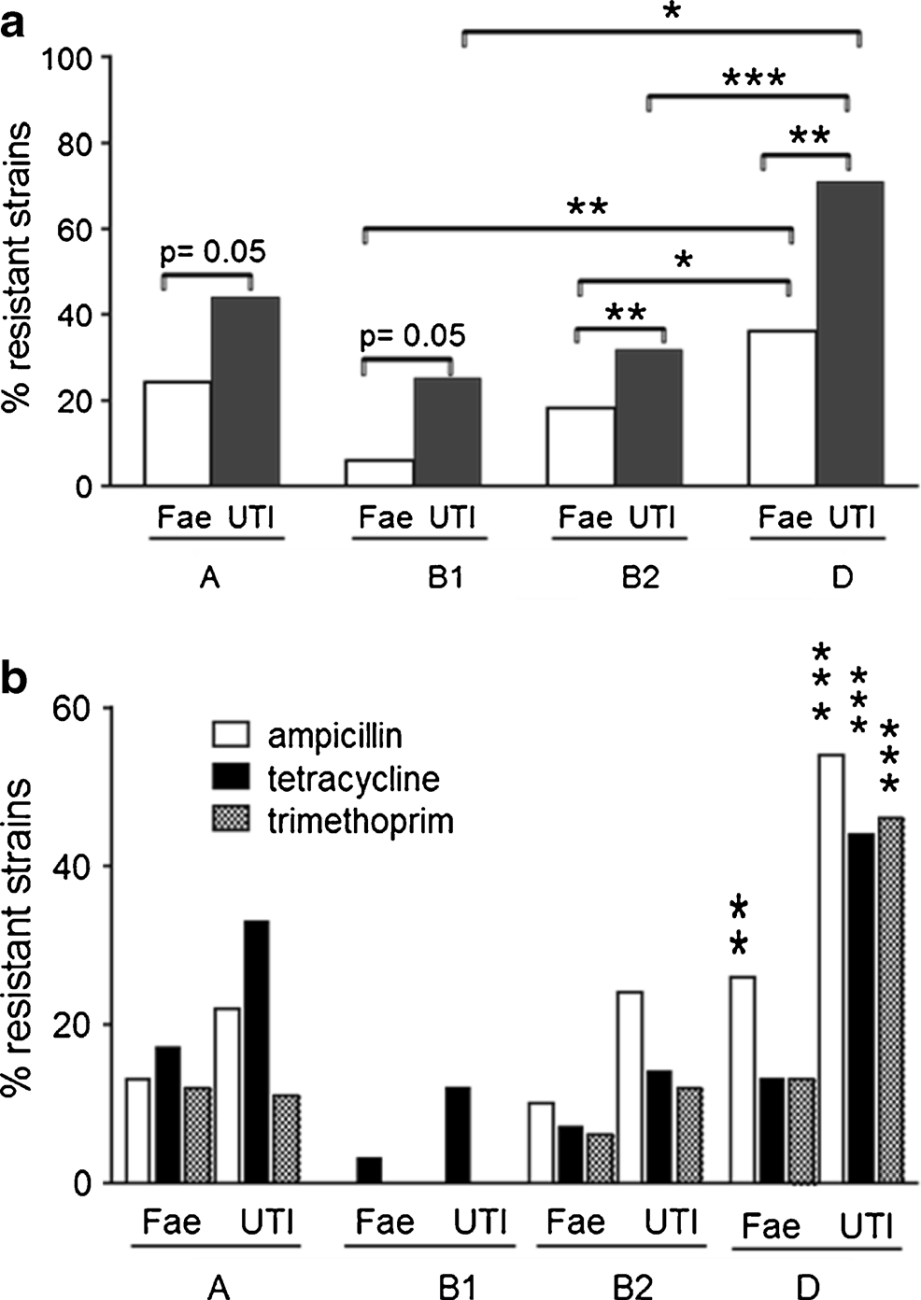
Antibiotic resistance in relation to phylogenetic group origin. **a** Prevalence of resistance to any of the screened antibiotics among fecal (*Fae*) and urinary (*UTI*) isolates as a function of phylogenetic group. **b** Resistance to ampicillin (*white bars*), tetracycline (*black bars*), and trimethoprim (*checkered bars*) as a function of phylogenetic group and clinical origin. The *asterisks* denote significance comparing the resistance of group D fecal or urinary strains to the other phylogenetic groups combined (Fisher’s exact test)

Resistance was more common among urinary than among fecal strains within each phylogenetic group, the difference being highly significant for groups B2 and D strains (*p* = 0.009 and *p* = 0.003, respectively) and of borderline sig-nificance for groups B1 and A strains (*p* = 0.05 for both). Furthermore, regardless of clinical origin, group D strains were significantly more often resistant than groups B2 and B1 strains (urinary strains: *p* < 0.0001, *p* = 0.04; fecal strains: *p* = 0.03, *p* = 0.004) (Fig. 1a).

Figure 1b shows resistance to individual antibiotics in relation to phylogenetic group origin. Urinary group D strains were most often resistant to each of the antibiotics (*p* ≤ 0.0001 for each, compared to urinary strains of the other groups combined) and fecal group D strains were more often resistant to ampicillin than fecal strains of the other phylogenetic groups (26 vs. 9%, *p* = 0.004).

As evident from Fig. 1b, ampicillin resistance was the most common resistance type in groups B2 and D strains, regardless of origin, while groups A and B1 strains were more often resistant to tetracycline than the other two antibiotics. Hence, resistance to ampicillin was significantly more common in strains belonging to groups B2 and D (combined) than in strains belonging to groups A and B1 (combined), 23 vs. 11%, *p* = 0.001. In contrast, resistance to tetracycline was equally common in strains from phylogenetic groups A and B1 (combined) and from phylogenetic groups B2 and D (combined) (15 vs. 17%, NS).

### Antibiotic resistance and virulence factor genes

UTI strains more often than fecal strains carried all tested virulence genes, i.e., *fimA* (74 vs. 73%, *p* = 0.92), *papC* (72 vs. 29%, *p* < 0.0001), *sfaD*/*E* (45 vs. 27%, *p* < 0.0001), *hlyA* (45 vs. 22%, *p* < 0.0001), *iutA* (56 vs. 28%, *p* < 0.0001), *neuB* (31 vs. 22%, *p* = 0.03), and *kfiC* (15 vs. 7%, *p* = 0.009). Furthermore, the two virulence factors *papC* and *iutA* were both significantly more common among UTI strains as compared with fecal strains within the same phylogenetic group (*papC*: *p* < 0.0001, *iutA*: *p* = 0.007 in group B2 strains; *papC*: *p* = 0.0009, *iutA*: *p* < 0.0001 in group D strains).

**Fig. 2:**
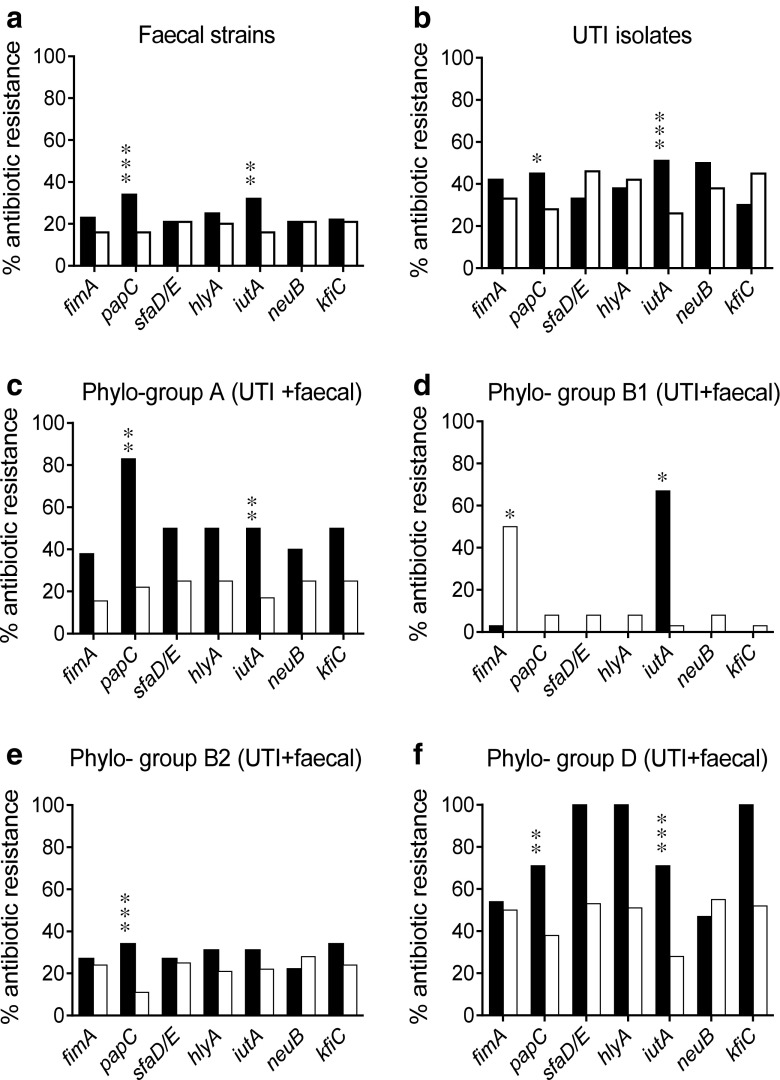
Antibiotic resistance in relation to virulence gene carriage. The prevalence (% of isolates) of resistance to at least one of the screened antibiotics is shown in isolates with (*black bars*) or without (*white bars*) seven virulence genes among fecal (**a**) and urinary (**b**) *E. coli* strains or in strains belonging to the four phylogenetic groups (**c**–**f**); in this case, both urinary and fecal strains were included. *p < 0.05, **p < 0.01, and ***p < 0.001 (Fisher’s exact test)

The relation between antibiotic resistance and virulence gene carriage is shown in Fig. 2. In both fecal (Fig. 2a) and UTI strains (Fig. 2b), strains carrying *papC* or *iutA* were more often resistant to at least one of the tested antibiotics than were strains lacking these traits. Figure 2c–f shows the association between antibiotic resistance and virulence gene carriage within each phylogenetic group, including both fecal and urinary strains in the analyses. Carriage of *papC* was significantly more common among resistant than susceptible strains within phylogenetic groups A (Fig. 2c), B2 (Fig. 2e), and D (Fig. 2f). Further, carriage of the aerobactin gene, *iutA*, was significantly more common in resistant than susceptible strains within the A, B1, and D phylogenetic groups (Fig. 2c, d, f).

### Prevalence of the CGA-D-ST69, O15:H1-D-ST393, and O25b:H4-B2-ST131 clones

Among urinary *E. coli*, 24 strains (12%) belonged to the CGA-D-ST69 clone, of which more than 75% carried *iutA* and *papC*. Among the fecal strains, 3 (1%) belonged to this clone, two of which carried *fimA*, *papC*, and *iutA*, and the third one only *fimA*. Fifty percent of the urinary CGA-D-ST69 isolates were resistant to at least three antibiotics, i.e., ampicillin (58%), tetracycline (58%), and trimethoprim (54%). Among the fecal CGA-D-ST69 strains, two were resistant to ampicillin and trimethoprim, and one was susceptible to all the antibiotics tested. Resistance to at least one of the screened antibiotics was significantly more common among urinary CGA-D-ST69 strains than among other urinary strains (75 vs. 35%, *p* = 0.0003). As this clone is known to be resistant to sulfonamide and streptomycin, we tested resistance to these antibiotics and found 63 and 38% to be resistant, respectively [14].

Only one (0.5%) of the urinary strains and two (1%) of the fecal strains belonged to the O15:H1-D-ST393 clone. All carried the *iutA* gene, and two carried *papC*. Two of three strains were each resistant to nalidixic acid and tetracycline, and the third fecal strain acquired a resistance plasmid during its colonization in an infant’s gut, as described elsewhere [10].

The O25b:H4-B2-ST131 clone that belongs to phylogenetic group B2 was represented by two urinary strains (1.0%) and one fecal (0.4%) strain. All three carried *iutA* but were fully susceptible to all the antibiotics tested.

### Multiple regression analysis

As seen in Table 2, phylogenetic group D origin was a strong independent predictor of being resistant to any of the screened antibiotics, as well as for resistance to ampicillin or trimethoprim. Carriage of *papC* and *iutA* were also strong predictors for resistance to any antibiotic, *papC* was also an independent predictor for resistance to ampicillin, and *iutA* was an independent predictor for resistance to both ampicillin and tetracycline. UTI origin was not an independent explanatory factor for antibiotic resistance. CGA-D-ST69 clonal origin was significantly associated with resistance to tetracycline (*p* = 0.03) and tended to be associated with trimethoprim resistance (*p* = 0.07, Table 2).

**Table 2:** Multiple regression analysis examining phylogenetic group, clinical origin, and virulence as determinants of resistance to any antibiotic, ampicillin resistance, tetracycline resistance, or trimethoprim resistance

	Resistance to:							
	Any antibiotic^a^		Ampicillin		Tetracycline		Trimethoprim	
	B^b^	*p*-Value^c^	B^b^	*p*-Value^*c*^	B^b^	*p*-Value^c^	B^b^	*p*-Value^c^
Intercept^d^	−1.53		−0.77		−0.30		−0.33	
Group D	1.02	**0.002**	1.04	**0.004**	0.36	0.39	0.90	**0.03**
*papC*	0.87	**0.0001**	0.78	**0.006**	0.47	0.88	0.36	0.29
*iutA*	0.74	**0.001**	0.63	**0.02**	0.98	**0.001**	0.62	0.06
UTI origin	0.30	0.23	0.57	0.05	0.34	0.28	0.45	0.19
CGA-D-ST69	0.66	0.23	0.48	0.36	1.23	**0.03**	1.01	0.07

^a^Resistance to the following antibiotics were included in the screening: ampicillin, cefuroxime, cefoxitin, mecillinam, cefadroxil, ceftazidime, chloramphenicol, gentamicin, tobramycin, nitrofurantoin, nalidixic acid, tetracycline, and trimethoprim

^b^B: standardized regression coefficient

^c^Bold *p*-value ≥ 0.05, statistically significant association

^d^Intercept: the point at which the curve intersects the *y*-axis

### Relation of resistance to population levels and time of persistence of *E. coli* strains in the microbiota

**Fig. 3:**
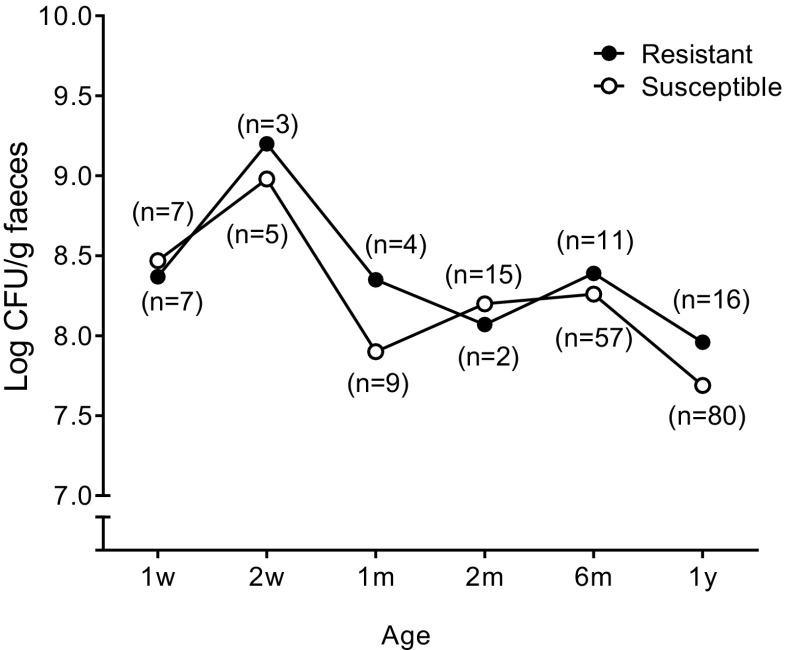
Fecal population counts of resistant and fully susceptible *E. coli* strains in infants’ samples. Strains exhibiting resistance to at least one antibiotic (*filled circles*) or strains susceptible to all screened antibiotics (*open circles*) derived from infants followed with quantitative stool cultures from 1 week to 1 year of age. The average counts of all resistant and susceptible strains that were isolated for the first time at each time point are presented. The numbers within parentheses denote the number of strains represented on each sampling occasion

Figure 3 shows the population levels at different time points after birth of strains that were resistant to at least one antibiotic and fully susceptible strains. At each time point, population counts for all strains that were isolated for the first time at that particular time point were included in the analysis (Fig. 3). There were no significant differences in population levels between resistant and fully susceptible strains on any sampling occasion. Because of the low number of strains resistant to ampicillin, tetracycline, or trimethoprim at each individual time point, the comparison to susceptible strains was not performed for strains resistant to individual antibiotics.

A part of the fecal strains had previously been characterized with respect to their period of colonization in the commensal microbiota [6]. Resident strains often belong to phylogenetic group B2 [4] and carry the *papC* and *iutA* operons [6, 7]. We included these factors, as well as a phylogenetic group D origin, as explanatory factors in a multiple regression analysis, to determine their contribution to antibiotic resistance. As shown in Table 3, being resident in the microbiota did not contribute to being resistant among the examined fecal strains. Belonging to group B2 was a significant negative predictor of antibiotic resistance, while *papC* was a significant positive predictor (Table 3).

**Table 3:** Multiple regression analysis examining the role of phylogenetic groups, virulence genes, and capacity to persist in the microbiota as determinants of antibiotic resistance among fecal commensal *E. coli* strains from infants

	Resistance^a^	
	B^b^	*p*-Value^c^
Intercept^d^	0.10	
Phylogenetic group D	0.5	0.42
Phylogenetic group B2	−1.3	0.04
*papC*	0.65	0.04
*iutA*	0.71	0.12
Resident strains^e^	−0.20	0.74

^a^Resistance to any of the antibiotics listed in Tables 1 and 2 was the dependent variable

^b^B: standardized regression coefficient

^c^Bold *p*-value ≥ 0.05, statistically significant association

^d^Intercept: the point at which the curve intersects the *y*-axis

^e^Ninety-five resident and 22 transient fecal *E. coli* strains were included in the analysis. Resident strains were those isolated repeatedly from an infant over a period of at least 3 weeks, while transient strains colonized for less than 3 weeks

## Discussion

In the present study, we investigated determinants of resistance to commonly used antibiotics in fecal and urinary *E. coli* strains from Swedish infants and young children below 2 years of age, and examined the relation between antibiotic resistance, virulence gene carriage, and phylogenetic group origin of the strains. In addition, we screened the strains to identify three known uropathogenic clones, i.e., CGA-D-ST69, O15:H1-D-ST393, and O25b:H4-B2-ST131, to determine if these clones contributed to the pool of antibiotic-resistant *E. coli* colonizing and infecting Swedish infants. In the multivariate analysis, we found three factors that contributed independently to the strains being resistant to at least one of the screened antibiotics; belonging to phylogenetic group D, and carrying the two UTI virulence genes, *papC* encoding P fimbriae and *iutA* encoding aerobactin. Neither persistence in the microbiota nor attaining high fecal population counts were associated with antibiotic resistance among the fecal strains.

Among the tested antibiotics, resistance to ampicillin, tetracycline, or trimethoprim were most common, but the prevalence was moderate compared to the reported figures from many other countries during the same period [23, 24]. This is in agreement with a generally low resistance rate among Swedish clinical isolates observed in surveillance studies (http://www.ecdc.europa.eu). Still, the 21% resistance rate among fecal commensal strains found here is considerably higher than the 14% reported in a Swedish study from the 1970s [25].

Resistance to tested antibiotics was significantly more common among urinary than among fecal strains. This is in accordance with other studies, showing that the frequency of resistance in commensal *E. coli* is lower but correlated to the resistance rate in clinical isolates from the same area [26]. However, clinical origin as a factor associated with resistance disappeared in the multivariate analysis, indicating that phylogenetic group origin and virulence profile were the primary explanatory factors. It is well known that *papC* and *iutA* are accumulated in uropathogenic strains.

One factor promoting antibiotic resistance development would be a pronounced capacity to pick up resistance elements transferred horizontally. Phylogenetic group D origin was a significant independent determinant of antibiotic resistance, in agreement with previous studies showing that a group D phylogenetic background *per se* facilitates acquisition of resistance genes [27, 28]. In accordance, we have reported on a case of transfer of a resistance plasmid from one group D strain to another in the intestinal microbiota of a child, in relation to treatment with ampicillin, to which the donor strain was resistant [10].

CGA-D-ST69 is a clone that often exhibits multiresistance due to a large conjugative plasmid containing several resistance genes [13]. The CGA-D-ST69 isolates were the most resistant ones, although fecal CGA-D-ST69 isolates were less resistant than urinary isolates of this clone. A CGA-D-ST69 clonal origin was an independent explanatory factor for resistance to tetracycline and tended to be an explanatory factor for resistance to trimethoprim. Notably, only 1% of the fecal strains, but 12% of the urinary isolates, belonged to this clone.

Almost 50% of the infantile fecal *E. coli* strains examined here belonged to phylogenetic group B2, a figure that we have reported previously [4]. *Escherichia coli* group B2 strains are the most pathogenic, but had overall low antibiotic resistance both in the urinary isolate collection and among the fecal strains. Indeed, a phylogenetic group B2 origin was a significant negative predictor of antibiotic resistance among fecal strains in the present study. The fact that B2 strains are less likely to carry resistance genes compared to group D strains confirms observations from previous studies [29]. Group B2 strains may be less prone to acquiring plasmids than group D strains. Accordingly, virulence genes in group B2 strains are mostly carried on pathogenicity islands located in the chromosome, whereas group D strains often carry virulence genes, e.g., *iutA*, on plasmids [29]. However, group B2 is highly genetically diverse, comprising at least nine subgroups [30], some of which may be better at incorporating horizontally transferred genetic elements than others. One of the most pathogenic clones of phylogenetic group B2 is O25b:H4-B2-ST131, which is enriched in virulence factor genes and, nowadays, frequently carries resistance genes encoding, e.g., CTX-M, KPC, or NDM enzymes [31]. Co-selection of resistance genes, e.g., CTX-M, and virulence genes in plasmids of the IncFII group was suggested to be of importance for the worldwide dissemination of this clone [32]. However, the three strains of this clone identified in the present study were sensitive to all the antibiotics tested. Our low isolation rate and lack of resistance among ST131 isolates likely reflect a low prevalence of this clone in Sweden before 2005, and an overall low resistance rate in *E. coli*. The prevalence of ESBL-producing *E. coli* was only 0.2% among urine isolates in 2004 in Western Sweden and only 10% of these isolates belonged to the ST131 clone [33].

Carriage of either of the virulence genes *papC* and *iutA* were independent explanatory factors for antibiotic resistance, including any resistance or resistance to ampicillin. *iutA* was also an explanatory factor for tetracycline resistance. The *pap* operon encodes P fimbriae, which enables adherence to both urinary and colonic epithelial cells and favors both persistence in the gut microbiota and urinary tract virulence [6, 7]. Aerobactin production, encoded by the *iutA* operon, is also linked to both urinary virulence [34] and persistence in the commensal gut microbiota [7]. Long-term persistence in the microbiota could enhance the chances of acquiring resistance genes and high population counts could also favor gene transfer. However, we could not demonstrate any independent contribution of long-term persistence in the microbiota on antibiotic resistance, nor did we observe any relation between fecal population counts and resistance.

Possibly, resistance genes and virulence genes occur together on transposons or plasmids and are, therefore, co-selected. Carriage of the *iutA* gene has previously been associated with antibiotic resistance in *E. coli* of extraintestinal origin [34] and is also common among strains producing ESBLs of the CTX-M types [35, 36]. In previous studies, we observed positive associations between carriage of *tet* genes encoding tetracycline resistance and the virulence genes *papC* and *iutA*, as well as between carriage of beta-lactamase genes and *papC* in commensal *E. coli* strains [37, 38]. In contrast, carriage of *pap* and *hly* genes is inversely related to resistance against fluoroquinolones in *E. coli*, possibly relating to a partial loss of genomic pathogenicity islands as a consequence of resistance mutations in genes encoding topoisomerases II and IV [39]. As few strains in our material were resistant to quinolones, this potential association could not be examined here.

In conclusion, we confirmed that strains belonging to phylogenetic group D have a special propensity to be resistant to antibiotics and showed that carriage of the virulence genes *papC* and *iutA* were also linked to resistance. The reason for the latter observation remains to be explained.
